# Criterion validity of five open-source app-based cognitive and sensory tasks in an Australian adult life course sample aged 18 to 82: Labs without walls

**DOI:** 10.3758/s13428-024-02583-1

**Published:** 2025-01-22

**Authors:** Shally Zhou, Brooke Brady, Kaarin J. Anstey

**Affiliations:** 1 School of Psychology, University of New South Wales, Sydney, Australia; 2 UNSW Ageing Futures Institute, Sydney, Australia; 3 Neuroscience Research Australia, Margarete Ainsworth Building, 139 Barker Street, Randwick, NSW 2031 Australia

**Keywords:** mHealth, Mobile research application, iOS, Psychometric testing

## Abstract

With recent technical advances, many cognitive and sensory tasks have been adapted for smartphone testing. This study aimed to assess the criterion validity of a subset of self-administered, open-source app-based cognitive and sensory tasks by comparing test performance to lab-based alternatives. An in-person baseline was completed by 43 participants (aged 21 to 82) from the larger Labs without Walls project (Brady et al., 2023) to compare the self-administered, app-based tasks with researcher-administered equivalents. 4 preset tasks sourced from Apple’s ResearchKit (Spatial Memory, Trail Making Test, Stroop Test, and dBHL Tone Audiometry) and 1 custom-built task (Ishihara Color Deficiency Test) were compared. All tasks except the Spatial Memory task demonstrated high comparability to the researcher-administered version. Specifically, the Trail Making Tests were strongly correlated (.77 and .78 for parts A and B, respectively), Stroop correlations ranged from .77 to .89 and the Ishihara tasks were moderately correlated (r = .69). ICCs for the Audiometry task ranged from .56 to .96 (Moderate to Excellent) with 83% sensitivity and 100% specificity. Bland–Altman plots revealed a mean bias between -5.35 to 9.67 dB for each ear and frequency with an overall bias of 3.02 and 1.98 for the left and right ears, respectively, within the minimum testing interval. Furthermore, all app-based tasks were significantly correlated with age. These results offer preliminary evidence of the validity of four open-source cognitive and sensory tasks with implications for effective remote testing in non-lab settings.

**Supplementary information:**

The online version contains supplementary material available at 10.3758/s13428-024-02583-1.

## Introduction

There has been a rapid increase in online and digital research studies conducted through smartphone apps (Koo & Vizer, 2019), further encouraged by technological advances and the impact of COVID-19. The two decades since 2000 saw an exponential rise in mobile health (mHealth) research with no trend of slowing down (Cao et al., 2021). As a result, there is a greater need for reliable and valid app-based tasks that can remotely assess various cognitive and sensory domains. Although there are many online assessments developed for computers, tablets and smartphones, not all tasks reporting psychometric properties are available for research and clinical use (see Amanzadeh et al., In Press; Bright & Pallawela, 2016; Chan et al., 2021 for systematic reviews). Furthermore, validation studies of computer and tablet cognitive assessments were more common than newer platforms such as smartphones (Öhman et al., 2021), highlighting a need for more smartphone-based validation studies.

When conducting remote observational studies, smartphone assessments overcome many of the limitations and barriers introduced by other digital platforms such as computers and tablets. Device preferences vary according to the testing context (clinic vs. remote), administration (participant vs. researcher) and whether studies provide a device or use participants’ personal device (Öhman et al., 2021). Recent reviews found studies in clinics were mostly completed on tablets whereas self-administered remote or home-based testing mostly used smartphones (Koo & Vizer, 2019; Öhman et al., 2021). Although computer- and tablet-based assessments are a closer replication of laboratory and clinical conditions within participants’ homes, they do not offer the same level of portability and convenience to participants and researchers when managing the assessment schedule (Harari et al., 2017). This is especially important to consider for studies that require high-frequency assessments.

Smartphone research apps have several benefits for studies that require more intensive measurement schedules and can increase the ecological validity of a project. For example, mHealth apps can take advantage of inbuilt smartphone sensors such as microphones, touchscreens, GPS, and accelerometer data enabling novel data that can be easily collected during participants’ day-to-day activities. The increased portability of research apps also gives researchers access to a wider range of participants including those living in regional or remote areas and those with health and mobility limitations rather than only targeting those who live nearby or who can travel to the lab for testing. Further, research apps can better manage the assessment schedule through sending notifications and reminders which increase response rate (van Berkel et al., 2020). Therefore, smartphone research apps are useful for studying a broader range of participants within real-life environments.

### Existing validated digital assessments

Existing validation studies of digital cognitive tasks demonstrate performance comparable to traditional neuropsychological tests. A meta-analysis of nine studies found significant correlations between mobile-based and validated traditional neuropsychological assessments such as the Trail Making Test (TMT), Stroop Test, and Mini-Mental State Exam (Siddi et al., 2021). Interestingly, each of those identified studies focused on one or a small number of assessments highlighting the need for a more comprehensive smartphone-based testing platform for cognitive and sensory tasks. Though there are some validated open-source cognitive tests for computers (e.g., Adolphe et al., 2022), to our knowledge, no studies have validated any open-source cognitive and sensory smartphone-based tasks.

As mentioned earlier, there are more comprehensive testing platforms adapted for computers and tablets than smartphones. Two prominent examples of validated cognitive assessments platforms are the NIH Toolbox® (Toolbox Assessments Inc, 2023b) and CANTAB® (Cambridge Cognition, 2023a) which are available for iPad and web-based testing. However, the NIH Toolbox® requires a researcher to administer tasks through an iPad and has yet to enable self-testing. Furthermore, at the time of writing, these tasks are yet to be fully translated for smartphones to enable remote testing. Cambridge Cognition have begun to roll out a mobile app for high-frequency smartphone-based testing (Cambridge Cognition, 2023b) and the NIA have recently funded development for a Mobile Toolbox expected to be released in 2024 (Gershon et al., 2022; Toolbox Assessments Inc, 2023a). Therefore, existing smartphone-based assessments are more limited in their options and use for remote research studies.

Another limitation of existing smartphone assessment platforms is that they have yet to enable the integration of surveys and sensor-based measures through their app. Without the source stimuli and code, these assessment apps would not be suitable for most research studies as they currently stand, especially for large longitudinal projects exploring multiple physical, psychological, cognitive, and sensory measures. Currently, to use the well-validated tasks from these assessment platforms, participants and researchers would need to have separate apps, or even digital platforms (e.g., combining smartphone with web-based or tablet-based measures), to administer all the elements of their project thereby increasing participant and research administrative burden. This gap in existing digital smartphone assessment options highlights a limitation that impacts the administration of remote psychological and mHealth studies and the need for more validated, open-sourced cognitive and sensory assessments.

### ResearchKit

Apple ResearchKit (Apple Inc, 2023a) is an opensource framework for building custom research apps that can implement all stages from onboarding and consent to data collection with integrations from smartphone and smartwatch sensors. Using ResearchKit, developers can create project-specific apps with surveys and tasks that can be downloaded onto participants’ iPhones. Allowing participants to use their own devices reduces research costs, increases accessibility, and avoids additional participant burden in having to learn to use and manage another device.

Developers have successfully used ResearchKit to create mHealth research apps with standardized presentation of questionnaires, cognitive and sensory tasks (for example, Bot et al., 2016; Chan et al., 2017; Egger et al., 2018). App-developers can create their own task or use predefined tasks from an existing library. However, tasks from this library have yet to be validated against in-person, researcher-administered tests that measured the same underlying constructs.

### Current study

The present study aimed to validate 5 app-based tasks, 4 currently available in the open-source ResearchKit Active Task library (Apple Inc, 2023c) and a new app-based version of the Ishihara color deficiency test developed by the study team with code made available on request to the study PI (Ishihara, 1917). Performance in the self-administered app-based tasks were compared to scores in their respective researcher-administered versions to evaluate criterion validity. It was hypothesized that all app-based tasks would be valid and comparable to their researcher-administered equivalent. Specifically, tasks would have ICCs and Pearson’s correlations of at least 0.5 and the dBHL Tone Audiometry would also show good sensitivity and specificity as demonstrated by a sum of at least 1.5, based on Power et al. (2013). Further, we expected all tasks aside from the Ishihara test to be at least moderately correlated with age.

## Methods

The current study is part of a larger project, Labs without Walls (see Brady et al., 2023 for the protocol), that investigated short-term changes in self-perceptions of ageing and gender among a life course sample across an 8-week study period. The purpose-built Labs Without Walls research app and paired Apple Watch devices were used to collect a range of data, including performance on app-based cognitive and sensory tasks.

### Participants

In the larger study, participants were recruited from across Australia between May 2021 – January 2023. Contact details were received through an online expression of interest webform, volunteer registries, volunteer job boards, email newsletters and social media callouts. Participants aged 18–85 years old, able to communicate in English, who owned an iPhone 6S or newer, and were willing to download the purpose-built free research application were eligible. To ensure an even distribution of participants across the life course by age and sex, minimum sample sizes were required for seven age categories and stratified by male and female sex assigned at birth.

This paper presents cross-sectional data from a subsample of 43 participants who were invited to complete an in-person onboarding session. Sessions were held either in participants’ homes across Sydney or at Neuroscience Research Australia (NeuRA) following COVID-safe protocols. The final sample included 18 men, 24 women and 1 non-binary person, aged 21 to 82 years (M = 51.57, SD = 19.93; excluding 1 participant who did not report their age, ethnicity, or education; Supplementary Table 1). All participants completed secondary school (mean years of formal education was 18.52 years, SD = 3.43). Most participants self-identified as Caucasian/White (n = 33) or East Asian (n = 6) and three South-East or West Asian. Informed consent was obtained from all participants and the research was approved by the UNSW Human Research Ethics Committee (HC200792).

#### Sample size calculations

A priori power calculations for this validation study were conducted in G*Power (version 3.1.9.7; Faul et al., 2009). To detect a moderate-to-large Pearson’s correlation based on Cohen’s effect sizes (r = 0.5; power = 0.8, alpha = 0.05, two-tailed), a minimum of 29 participants was needed.

Validity of the Audiometry was assessed through intra-class correlations (ICC). Sample size was calculated in R (version 4.1.1; R Core Team, 2021) in RStudio (version 2023.06.0.421; Posit Team, 2023) with the ICC.Sample.Size package (Rathbone et al., 2015) based off the methods derived by Zou (2012). A minimum of 28 participants was needed to detect an ICC of 0.5 (power = 0.8, alpha = 0.05, two-tailed). This effect size was chosen as anything below this value is considered poor reliability (Koo & Li, 2016).

Previous systematic reviews of high-frequency Ecological Momentary Assessment study compliance and attrition rates found that compliance fell in studies with longer durations and more frequent assessments (Wrzus & Neubauer, 2023). Therefore, to account for possible missing data and attrition over the 8-week study, a minimum of 40 participants was recruited.

### Procedure

The 2-h onboarding session involved providing informed consent through the research application, setting up study devices, and completing a series of cognitive and sensory tasks with a trained researcher. All tasks were delivered following a standardized protocol. Participants who travelled to NeuRA received a $20 gift card to contribute to travel costs. All participants were provided with Apple wired EarPods (model number MMTN2FE/A) to standardize the administration of the app-based dBHL Tone Audiometry Task.

### Labs without walls App

App-based assessments were delivered through the Labs without Walls research app, available through the App Store for all iPhones using iOS 13 and later. The app was primarily built using Swift (Apple Inc) within the XCode (Apple Inc) development environment with Objective-C (Apple Inc) to extend the ResearchKit framework (Apple Inc, 2023a; see Brady et al., 2023 for more detail). ResearchKit was used for features including consent forms, surveys, prebuilt active tasks and summary graphs. With participant consent, the app had read-only access to the local health store through Apple’s HealthKit. Throughout the study, data were continuously gathered from the Apple Watch devices and iPhone. The app fully supported light and dark mode displays. Amazon Web Services was used to securely host the backend data collection. Access to data was restricted to only essential research staff approved by ethics with two-factor authentication and 6-monthly audits of data access. Identifiable participant information was stored separately from deidentified study data.

The code for all but one of the app-based tasks (Ishihara Color Deficiency Task) were predefined ‘active tasks’ available through ResearchKit. Code for the Ishihara color deficiency task is available through contacting the study PI (Anstey). Tasks were released on their scheduled day and remained in the task list for up to 3 days.

### Measures

#### Spatial memory

##### Lab-based assessment 

The forward span Corsi Block task from the Wechsler Memory Scale (3rd edition; Wechsler, 1997) was used to assess short-term spatial memory. Participants sat directly opposite the researcher with a board of nine blocks between them and must replicate a sequence tapped by the researcher. Responses were scored as 1 if participants correctly repeated the entire sequence and 0 for any other response. There were two trials for each sequence length starting from two blocks and trials continued until participants made an error on both trials of a sequence length. The final score was a total out of 16.

##### App-based assessment

**Fig. 1:**
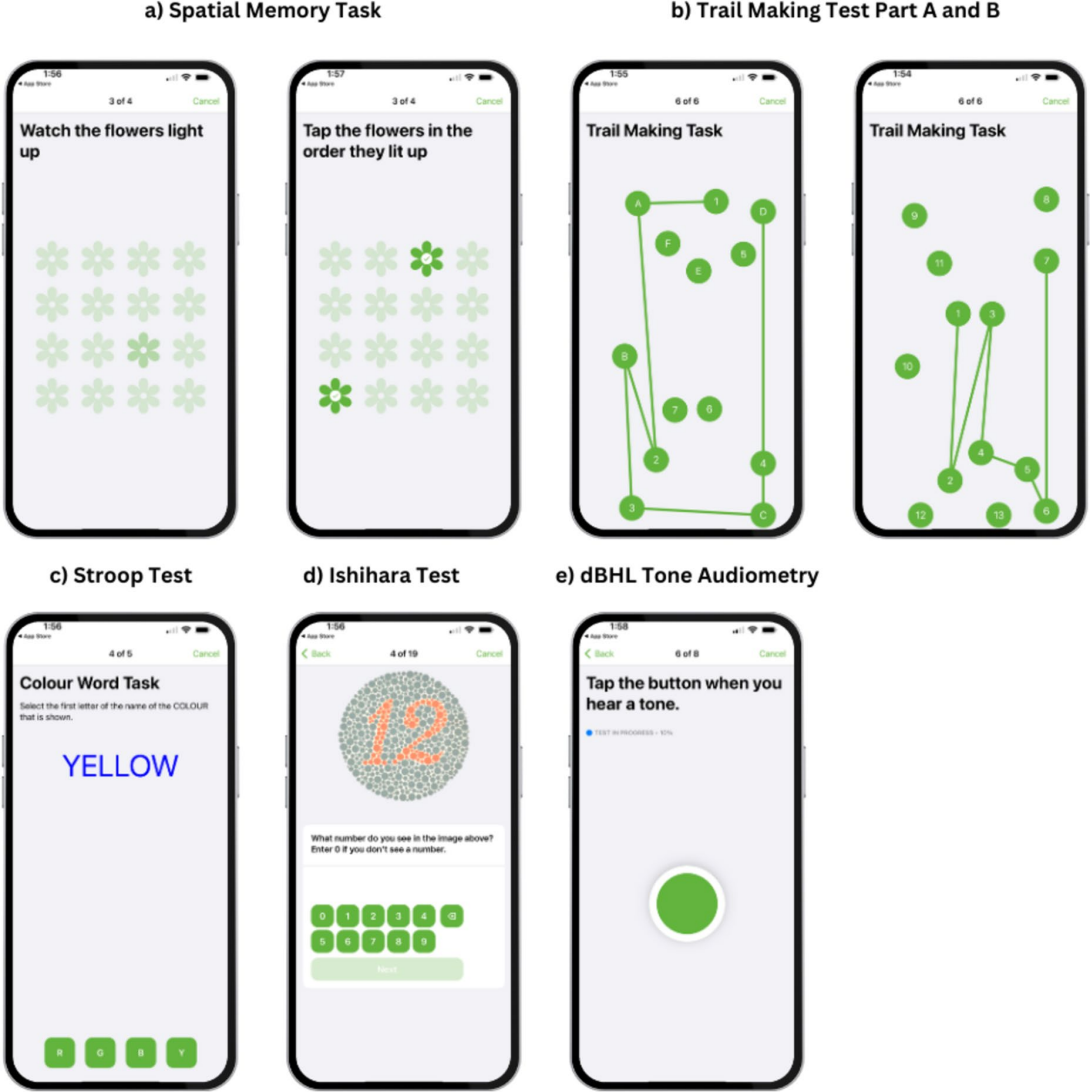
Example Screenshots from the App-based Cognitive and Sensory Tasks. Note. a) From the spatial memory task depicting the presentation of stimuli (left) and participant response (right). b) Trail Making Test Part A (left) and Part B (right). c) An example of a trial in the Stroop Task. d) a trial in the Ishihara Color Deficiency Test. e) Screenshot of the dBHL Tone Audiometry. Participants must press the button whenever they hear a tone

The spatial memory task also assessed short-term visuospatial memory and executive functioning in a game-like format (see Fig. 1a; Bot et al., 2016). For each trial, participants saw a sequence of flowers flash on a grid and had to reproduce it. Every correct response increased the sequence length by one. Sequence lengths started from 3 items shown on a 3 × 3 grid and moved to a 4 × 4 grid for sequences of 5 items or longer. The task ended when the participant reached 5 trials or failed 3 consecutive trials. The app calculated a default game ‘score’ ranging from 0–450 based on the number of correct responses within a sequence and the sequence length. To improve comparability with the lab-based task, the total number of correct trials was also calculated with scores ranging from 0–5.

#### Trail Making Test (TMT)

The Trail Making Test (TMT; Partington & Leiter, 1949) is a standard neuropsychological assessment with two distinct parts. Part A (TMT-A) measures sequencing, visual search, and processing speed whereas Part B (TMT-B) measures cognitive flexibility, alternating attention in addition to the skills required for TMT-A.

##### Lab-based assessment

A pen-and-paper Trail Making Test was administered. The researcher explained the instructions with a short practice trial before each test. In TMT-A, participants drew lines connecting 25 bubbles in order from 1 to 25 (e.g., 1–2–3…) as quickly as possible. In TMT-B, participants alternated between a number and a letter (e.g., 1-A-2-B-3-C…). Participants were to correct any errors and, if they did not notice the error, were immediately redirected to the last correct response by the researcher without pausing the timer. The total time and number of mistakes made were recorded for both parts.

##### App-based assessment

Similar to the pen-and-paper task, participants tapped a series of labelled bubbles in numerical order or alternated between a number and a letter (Fig. 1b). Instructions with images were provided without any time constraints. Once participants indicated they were ready to begin, there was a three second countdown before the task began. The task was coded so that bubbles were randomly generated across the screen and the trail path never crossed over a previous line. There was a total of 13 bubbles for each part (i.e., starting at 1 and finishing at either 13 for TMT-A and 7 for TMT-B). The total time and number of errors were recorded.

#### Stroop test

The Stroop task (Stroop, 1935) is commonly used to assess cognitive flexibility, executive function, and inhibition. There are many types of Stroop tasks and this study uses the color-word Stroop.

##### Lab-based assessment

The Victoria Stroop Task is a well-validated measure of executive function and inhibition with normative data for adults 18–94 years (Troyer et al., 2006). Three conditions were tested: dots, words, and color words. In the dots condition, participants were presented with a card and named the color of 24 dots as quickly as possible. In the words condition, participants saw 24 random words written in colored ink and named the color of the ink. The color words condition was similar to the word condition, but the words were one of the four colors presented. The total time and number of mistakes was recorded. The interference score was the time for the color-word divided by the time for the dots condition.

##### App-based assessment

Participants were shown colored words individually and selected the color of the ink from the buttons at the bottom of the screen. The response options were the first letter of each of the color options (i.e., “r” for “red”; Fig. 1c) Congruent trials occurred when the color of the ink matched the word (e.g., “green” written in green ink). Incongruent trials occurred when the ink did not match the word (e.g., “green” written in blue ink). There was a total of 48 trials, evenly split between congruent and incongruent trials, presented in a randomized order. The total number of correct responses and average trial time for each condition was recorded. The interference score was the difference between incongruent and congruent trial times.

#### Ishihara Color Deficiency test

The Ishihara Color Deficiency test (Ishihara, 1917) primarily assesses red-green color deficiency. The original task involves showing participants pseudo-isochromatic plates from a handbook of 38. Each pseudo-isochromatic plate contained a series of dots of various sizes and colors that form digits visible against the background of dots. Numbers are typically only perceived by those with normal color vision. However, the first (control) plate is designed to be visible to all participants and used as an example.

##### Lab-based assessment

The 25-plate version of the Ishihara color deficiency test was administered. Participants responded by identifying the number shown on the plate or stating they did not see a number. The first 21 plates are used to establish color deficiency and plates 22–25 to categorize the type of red-green color vision. The number ‘normal’ responses were summed and participants scoring 17 or more of the first 21 plates are regarded as having normal vision. Scores of 13 or less suggests red-green color deficiency which can be further categorized using plates 22–25.

##### App-based assessment 

Our developer created a smartphone version of the Ishihara color deficiency test as a ResearchKit survey (Fig. 1d). Participants were first asked to self-report any color-blindness before starting the task. The original Ishihara plates are freely available in the public domain for use as the copyright has expired in the US, Japan and Australia. The present task used images obtained online (*Ishihara Charts*, Harari et al., 2017). The 15 plates included 1 control plate and 14 test plates (plates: 1–13, 16 and 17). Failing 3 or more plates suggests possible deficiency and 5 or more errors suggests certain color deficiency based on Cole (2007).

### dBHL tone audiometry

Hearing thresholds were measured using an audiometer. Participants were asked to remove or turn off hearing aids and devices prior to starting the task.

#### Lab-based assessment

SHOEBOX Audiometry (SHOEBOX Limited, Ottawa, Ontario, Canada) is a tablet-based audiometer that has been clinically validated for diagnostic use (Saliba et al., 2017; Thompson et al., 2015). Thresholds were determined using a modified Hughson-Westlake method and calibrated transducers (RadioEar DD450). Test frequencies of 250, 500, 1000, 2000, 3000, 4000, and 8000 Hz were chosen to assess a broad range including values commonly used to assess hearing loss. Stimulus intensities ranged between 0 to 90 decibels.

Prior to starting the task, the researcher ran an ambient noise check to ensure suitable testing conditions. Participants wore transducers and sat facing away from the researcher and indicated whenever they heard a tone. The researcher presented trials by pressing a button for one second on the tablet every 3 to 5 seconds. Catch trials where no tones were delivered with the button press were also present throughout the assessment to ensure participants were responding to the tones rather than other environmental sounds.

#### App-based assessment

The dBHL Tone Audiometry task also used the Hughson-Westlake method to determine thresholds with Apple wired EarPods. Test frequencies matched those used for SHOEBOX Audiometry and intensities were predetermined by the task, ranging from −10 to 110 in 5 decibel increments.

First, an ambient noise check was performed using the iPhone’s internal microphone to ensure testing environments were under 45 dB. One-second tones were delivered randomly every 3–4 s and participants pressed a button whenever they heard a tone (Fig. 1e). Thresholds were recorded as 100,000 when participants did not respond to any of the tones presented for the frequency.

### Data analysis

Data were checked prior to analyses for quality and completion rates. The results of these checks are reported in the supplementary materials.

Statistical analyses and data visualizations were conducted using R (version 4.1.1; R Core Team, 2021) and RStudio (version 2023.06.0.421 Posit Team, 2023). Pearson’s correlation was used to establish criterion validity and determine whether performance in the self-administered app-based TMT, Spatial Memory, Stroop and Color Deficiency tasks were comparable to previously validated researcher-administered tasks. Pearson’s correlation was chosen as some tasks had minor differences in the length and scoring methods due to these tasks being adapted for high-frequency testing.

For tests of hearing function, audiometry thresholds were compared using ICC and Bland–Altman plots to quantify the agreement between tasks. ICCs were interpreted according to Koo and Li (2016) where values < 0.5 is considered poor, 0.5 to 0.75 is considered moderate, 0.75 to 0.9 is considered good, and > 0.9 is considered excellent correlation. Values of 100,000 were treated as missing and excluded from analyses. Sensitivity and specificity analyses were conducted by separating participants into two groups based on whether they may have mild hearing loss (i.e., a pure-tone average (PTA) of less than 25 dB). Based on Power et al. (2013), the threshold for a useful test is determined by having the sum of the sensitivity and specificity be at least 1.5. Positive and negative predictive values were also calculated to assess the likelihood of a ‘positive’ result in the self-administered app-based task being a genuine case of mild-hearing loss as measured by the researcher-administered task and a ‘negative’ result being a genuine case of normal hearing.

## Results

### Descriptive statistics

Table 1 presents the means, standard deviations and ranges for each of the researcher-administered and self-administered tasks except the audiometer data. Since age is often associated with cognitive and sensory functioning, Pearson’s correlations between tasks and chronological age are also displayed. Older age was associated with lower scores across both spatial memory tasks (r = 0.52, p = 0.004; r = 0.38, p = 0.019), slower reaction times for the app-based TMT (A: r = 0.37, p = 0.033; B: r = 0.47, p = 0.003) and researcher-administered TMT-B (r = 0.44, p = 0.006), slower responses and higher interference in the Victoria Stroop (r = 0.62, p < 0.001). Older participants also had significantly higher PTA thresholds across both the app-based and researcher-administered audiometers.

**Table 1:** Mean, standard deviations and Pearson’s correlations with age for each task

Task	N	Unit	Mean	SD	Range	Correlation with age	p-value
Corsi Blocks Forward-span score	43	Correct response	8.09	2.18	4 – 13	−0.52	0.004
TMT-A	43	Task time (seconds)	33.61	13.39	18.09 – 93.02	0.30	0.064
TMT-B	43	Task time (seconds)	80.61	62.67	29.20 – 333.00	0.44	0.006
Stroop dots	42	Task time (seconds)	14.09	4.32	8.07 – 32.82	0.17	0.315
Stroop words	42	Task time (seconds)	17.32	5.25	10.97 – 37.21	0.49	0.006
Stroop colored words	42	Task time (seconds)	27.02	13.18	14.98 – 94.41	0.51	0.001
Stroop interference	42	Ratio	1.93	0.60	1.10 – 3.65	0.62	< 0.001
25-plate Ishihara	43	Correct trials	19.81 (median = 21)	3.33	1 – 21	−0.17	0.072
SHOEBOX Audiometry	43	PTA (dB)	11.66	13.35	0.00 – 48.75	0.67	< 0.001
**Spatial Span score**	**38**	**Points**	**249.00**	**62.75**	**135 – 400**	**−0.38**	**0.019**
**TMT-A**	**36**	**Task time (seconds)**	**15.80**	**11.92**	**7.31 – 79.73**	**0.37**	**0.033**
**TMT-B**	**37**	**Task time (seconds)**	**20.98**	**13.04**	**10.30 – 84.8**	**0.47**	**0.003**
**Stroop match**	**37**	**Trial time (seconds)**	**1.24**	**0.31**	**0.81 – 2.32**	**0.12**	** < 0.001**
**Stroop mismatch**	**37**	**Trial time (seconds)**	**1.48**	**0.40**	**1.02 – 2.6**	**−0.24**	**0.005**
**15-plate Ishihara**	**42**	**Correct trials**	**13.71 (median = 15)**	**2.80**	**2 – 15**	**−0.29**	**0.015**
**dBHL Tone Audiometry**	**32**	**PTA (dB)**	**14.43**	**12.41**	**−5.00 – 48.75**	**0.65**	** < 0.001**

*Note*: self-administered, app-based tasks are bolded

Mean hearing thresholds for the SHOEBOX audiometer ranged from 9.42 to 31.05 dB in the left ear and 9.42 to 29.88 dB in the right ear when comparing across frequencies. For the app-based task, the mean threshold across frequencies ranged from 14.66 to 29.82 dB in the left ear and 13.21 to 26.35 dB in the right ear.

Pure Tone Averages in the better ear were calculated to identify participants with potential mild hearing loss (PTA > 25) from those with normal hearing. SHOEBOX identified 6 participants with mild hearing loss and 26 with normal hearing.

### Correlations between lab-based and app-based tasks

Pearson’s correlations between researcher-administered and self-administered tasks were all significant except for the spatial memory task. The 15-plate app-based Ishihara was positively correlated with the researcher-administered 25-plate version (r = 0.69, p < 0.001). TMT times were also strongly positively correlated for part A (r = 0.77, p < 0.001) and B (r = 0.78, p < 0.001). For the Stroop, the app-based ‘match’ condition was correlated with the dots (r = 0.89, p = 0.005) and words conditions (r = 0.87; p < 0.001) and ‘mismatch’ condition was comparable to the ‘color-words’ condition (r = 0.77, p < 0.001). The spatial memory task associations were not significant when comparing the app-based score with the Corsi total score (r = 0.175; p = 0.275), nor when comparing to maximum sequence lengths (r = 0.219; p = 0.203) or the number of correct trials across the two tasks (r = 0.09; p = 0.363).

### dBHL tone audiometry

App-based audiometry showed moderate to excellent validity comparing across frequencies and ear when excluding 100 000 values (ICC = 0.56 to 0.96, p < 0.001; Table 2). Bland–Altman plots visualize the differences between thresholds across each instrument by ear and frequency (see supplementary Figs. 1 and 2). The average bias ranged from −5.35 to 9.67 with an overall bias of 3.02 and 1.98 for the left and right ears, respectively (Table 3). All biases except the 250, 500 and 4000 Hz of the left ear were within 5 decibels (the testing interval) meaning the app-based audiometer reported slightly higher thresholds for lower frequencies than the SHOEBOX but nearly no difference for frequencies 2000 Hz and higher aside from 4000 Hz in the left ear. Overall sensitivity was 83% (5/6 participants with potential mild hearing loss correctly identified) and specificity was 100% (26/26 participants with normal hearing correctly identified). Positive and negative predictive values (i.e., the proportion of those identified with or without possible mild hearing loss as truly having mild hearing loss or normal hearing) comparing the app-based with the lab-based audiometer were 100% and 96.3%, respectively.

**Table 2:** Intra-class Correlations between SHOEBOX and App-based Audiometers by Ear and Frequency with 95% Confidence Intervals

Frequency (Hz)		Left	95% CI			Right	95% CI	
	n	ICC	Lower	Upper	n	ICC	Lower	Upper
250	29	0.69**	0.49	0.82	28	0.70**	0.51	0.83
500	30	0.56**	0.32	0.74	27	0.68**	0.48	0.81
1000	31	0.66**	0.45	0.80	29	0.82**	0.69	0.90
2000	28	0.84**	0.72	0.91	27	0.90**	0.82	0.94
3000	29	0.84**	0.73	0.91	27	0.92**	0.86	0.96
4000	28	0.90**	0.83	0.95	26	0.89**	0.80	0.94
8000	28	0.92**	0.86	0.96	26	0.96**	0.92	0.98

** *p* < 0.001

**Table 3:** Average Bias and 95% Limits of Agreement (LoA) from Bland–Altman Plots by Ear and Frequency

**Frequency (Hz)**		**Left**	**95% LoA**			**Right**	**95% LoA**	
	**n**	**Bias**	**Lower**	**Upper**	**n**	**Bias**	**Lower**	**Upper**
250	29	6.72	−10.31	23.75	28	3.57	−11.48	18.63
500	30	9.67	−14.74	34.07	27	5.00	−11.31	21.31
1000	31	5.00	−19.92	29.92	29	4.83	−10.33	19.98
2000	28	0.71	−18.47	19.89	27	3.89	−9.79	17.57
3000	29	1.03	−18.63	20.70	27	−0.74	−16.28	14.80
4000	28	−5.35	−23.43	12.72	26	−3.46	−23.98	17.05
8000	28	3.39	−17.45	24.23	26	0.77	−14.33	15.87

The app-based task tested −10 to 110 dB, whereas SHOEBOX tested 0 to 90 dB in 5 dB 554 increments

## Discussion

This study evaluated the criterion validity of five cognitive and sensory self-administered, app-based tasks against a researcher-administered equivalent in an Australian adult life course sample aged 18 to 82 years. Significant associations were found between performance on the app-based TMT, Ishihara, Stroop and dBHL Tone Audiometry tasks when compared to their lab-based alternatives. However, performance on the app-based spatial memory task was not significantly correlated with the Corsi Block Task despite both tasks being negatively correlated with age.

The correlations observed in the TMT and Stroop tasks were all comparable to previous validation studies (see Siddi et al., 2021 for a review). Stroop correlations were similar to other digital assessment validation studies (Jongstra et al., 2017). Similarly, correlations for TMT performance were comparable to another tablet-based validation in healthy young and older adults aged 19 to 82 years (Park & Schott, 2022) and smartphone TMT for healthy adults over 50 with parental history of dementia (Jongstra et al., 2017). Therefore, there is growing evidence that these brief self-administered smartphone tests are valid alternatives to the conventional researcher-administered tests.

The dBHL Tone Audiometry task had moderate to excellent ICCs ranging from 0.56 to 0.96, high sensitivity and specificity (83% and 100%, respectively) and high positive and negative predictive values. These results are comparable to other validations of self-administered and researcher-administered audiometry tasks. For example, the ICCs were comparable to other self-administered iOS audiometers that used commercially available earbuds such as the Apple wired EarPods (ICC = 0.57—0.97; Cunha et al., 2023; Kelkar et al., 2022). Mean biases from the Bland–Altman plot were mostly within 5 decibels, the minimum testing interval and the variability in mean biases is potentially driven by the up-to-20-decibel difference in the tested thresholds (−10 to 110 for app-based vs. 0 to 90 for SHOEBOX). Furthermore, the sensitivity and specificity observed in the present study were within the range found in other audiometry apps, especially those that also used standard Apple headphones or wired EarPods (Bright & Pallawela, 2016; Chen et al., 2021). This study is one of the first to contribute a validated open-source app-based audiometer to the literature. These results further support the growing evidence that self-administered app-based audiometry tasks can be valid alternatives to the researcher-administered audiometer and may be a viable option to measure hearing thresholds for non-clinical research.

The Corsi Block test and Spatial Memory Task may target slightly different cognitive and motor skills due to the gamification of the app-based task. Though both tasks assess visuo-spatial memory, the Corsi Block test may not have been an appropriate comparator as the Spatial Memory Task also assessed executive functioning (Apple Inc, 2023c). Gamified cognitive tasks have many benefits including increasing participant motivation and ecological validity but can introduce complexities such as a mixed-domain measure (Lumsden et al., 2016). In this app-based task, the flowers were evenly spaced on a flower grid whereas the nine Corsi blocks were unevenly distributed on a larger board. Additionally, the spatial memory task presented sequences of 5 or longer on a 4 × 4 grid rather than the 3 × 3 which includes more response options than the Corsi Block task. It’s unclear whether the differences in positions and numbers of flowers and blocks may increase cognitive load in memorizing the exact positions of items across the tasks. On the other hand, the app-based task may be prone to more accidental errors as it requires more fine-motor control to tap the correct flower on the smaller iPhone screen compared to the 1.25″ cubes on the Corsi board. Therefore, the Corsi Block test may not have been the most appropriate comparator for the app-based spatial memory task.

Another explanation for the non-significant correlation between the app-based spatial memory task and Corsi Block test relates to the testing method and settings of the task. As noted in the method section, the Corsi Block test had a binary score (0 or 1) for each trial whereas the app-based task assigned points for every correct response within the sequence of each trial. Secondly, the app-based spatial memory task, developed for high-frequency testing, only had 5 trials to minimize participant burden, and this may not have allowed sufficient variability across scores. However, there was no change in significance even when these analyses were re-run to compare the number of correct trials.

Although the spatial memory task did not significantly correlate with the Corsi Block test, they both had significant negative correlations with age, as predicted. The negative correlations with age indicate that both measures saw poorer performance among older participants and may still be informative in measuring participants’ overall cognition across the life course. However, the non-significant correlation between the two tasks may be due to tasks targeting distinct cognitive abilities that are not directly comparable.

## Limitations and future studies

There are limitations due to our sampling methods as the study was conducted throughout COVID-19 lockdowns and consisted of mostly Caucasian, highly educated participants from greater Sydney which may impact generalizability to the broader population. These results may not translate to underrepresented populations such as those living in rural areas, culturally and linguistically diverse populations or those with mobility or health concerns. Future studies are needed to validate these tasks among nationally representative and underrepresented samples.

Another consideration is the possible impact of sampling bias as ResearchKit tasks are only compatible with iPhone devices. Globally, Android users account for nearly 70% of mobile users however this trend is reversed in Australia with iOS users accounting for 60.8% of smartphone users, respectively (Statcounter, 2023). Future studies are needed to evaluate whether the testing platform (e.g., iOS vs Android) has any impact on the validity of app-based cognitive and sensory tests and seek to have app-based assessments accessible to multiple operating systems.

The temporal latency between researcher-administered tasks at baseline and the app-based tasks may also affect the current results. As participants completed some app-based tasks up to 40 days after the baseline, we must consider the effects of testing order, missing data, and contextual factors including daily variability in fatigue and mood. However, this design was chosen due to timing and budget constraints as shorter testing sessions were required under Sydney’s COVID-19 restrictions. Furthermore, some tasks are not expected to fluctuate between testing times. For example, results of the Ishihara Color Deficiency Test are not expected to change within a week and Australian legislation only require audiometric testing every 2 years among occupations with risk of hearing loss unless there are existing concerns or current hearing loss (SafeWork NSW, 2023). Furthermore, there was only a small effect of daily variability among participants completing cognitive tasks across 100 daily sessions and more variability was observed in younger than older adults (Schmiedek et al., 2013). Thus, the delay between app-based and lab-based testing is not expected to greatly impact any tasks.

Finally, the main trade-off with app-based studies is that researchers have less control over the testing environment in exchange for higher ecological validity. This is even more prominent with self-administered, remote research designs. Researchers can still include certain checks for validity and prompt participants with notifications and instructions. However, studies mostly rely on participants complying with study protocols. For example, the current hearing test included a background noise check prior to starting the task to control ambient noise in the testing environment. Nevertheless, as this was a self-completed task, researchers cannot confirm whether missing data in the task is due to participants not hearing presented tones or due to external causes or distractions. From the data alone, it is unclear whether there were any disturbances during tasks such as an incoming phone call or a fire alarm. These unexpected environmental differences may explain part of the variability seen in the ICCs and Pearson’s correlations. Future studies should consider the impact of testing environment on assessment results by allowing participants to report disturbances or unexpected interruptions when completing app-based tasks remotely.

It is also important to note that participants in this study used various iPhone models (iPhone 6S or newer) that could have impacted app-based task performance. Different iPhone generations vary in sensors, processing speed, device latencies (Nicosia et al., 2023) and screen sizes ranging from 4.7″ (iPhone 6S) up to 6.7″ (iPhone 14 Pro Max; Apple Inc, 2023b) which may affect the experience and performance in app-based tasks. For example, participant feedback indicated those with smaller screen sizes were unable to see all keys of the initial number keypad used in the Ishihara task (Brady et al., submitted). However, this error was promptly fixed and should not have impacted the overall task performance. Furthermore, the total latency across devices only ranged between 35 to 140 ms, which may not be important for tasks that do not require high precision in measuring response time (Nicosia et al., 2023). Therefore, researchers must also consider the potential impact of variability from study devices and whether they are within a reasonable range for the study aims.

## Conclusion

To our knowledge, this study is the first to demonstrate the validity of the TMT, Stroop, dBHL Audiometry and Ishihara Color Deficiency tasks available through the open-source Apple ResearchKit framework and administered on participants own iPhones. Labs without Walls also demonstrates the ease in creating a new sensory task and contributes the code and stimuli of a validated Ishihara Color Deficiency test. These are encouraging results for researchers to continue developing and using app-based assessments to measure cognitive and sensory functioning. In addition, the app-based dBHL Tone Audiometry task was psychometrically valid, providing a portable non-clinical hearing assessment using commercially available earbuds and freely available software through ResearchKit. Having these validated and open-source ResearchKit tasks would address the lack of an all-in-one smartphone research app that can collect multiple types of data from surveys, sensors, and cognitive and sensory tasks among participants using iOS devices. With the addition of validated tasks, ResearchKit can increase researchers’ potential for conducting remote psychological and mHealth studies especially those adopting intensive longitudinal designs.

## Supplementary information

Below is the link to the electronic supplementary material.

Supplementary file1 (DOCX 55 KB)

## Declarations

### Open practices statement

This study was pre-registered through OSF and can be found under the registrations tab in the main project folder (https://osf.io/3gk4f/). The materials for the app-based Ishihara task are freely available in the public domain (Commons) and the code for the app-based task is available on request to the study PI (Anstey). All other app-based tasks mentioned in the paper are freely available through Apple’s ResearchKit (http://researchkit.org/).

The datasets generated and analyzed during the current study are not publicly available as, according to the approved ethics protocol, participants did not consent to their data being shared outside the immediate research team without ethical approval. However, authors can be contacted with any questions related to the data and de-identified data are available on reasonable request with ethical approval. Because of this limitation, analysis code is illustrated with a synthetic dataset, which allows readers to check the correctness of their implementation. The analysis code can be accessed at https://osf.io/2nswx/.
