# Supplementary material for: Criterion validity of five open-source app-based cognitive and sensory tasks in an Australian adult life course sample aged 18 to 82: Labs without walls
Source: Behav Res Methods. 2025 Jan 22;57(2):69. doi: 10.3758/s13428-024-02583-1 (PMC11754352; doi:10.3758/s13428-024-02583-1)
Supplement: Supplementary file 1 — Supplementary file1 (DOCX 55 KB) [file 13428_2024_2583_MOESM1_ESM.docx]

Appendix

Participant demographics

**Supplementary Table 1.**

*Participant demographics of the in-person sample (N = 43).*

|  | Mean (SD)/ Number | Range/ % |
| --- | --- | --- |
| Age (years) | 51.57 (19.93) | 21 – 82 |
| Total Education (years) | 18.52 (3.43) | 12 – 30 |
| Gender |  |  |
| Male | 18 | 41.86 |
| Female | 24 | 55.81 |
| Non-binary | 1 | 2.33 |
| Ethnicity |  |  |
| Caucasian/White | 33 | 78.57 |
| East Asian | 6 | 14.29 |
| South-East Asian or West Asian | 3 | 7.14 |

Note: one participant did not report their age, ethnicity, or education.

Pre-analysis data checks

During the in-person testing, one participant with self-reported color blindness did not complete the Victoria Stroop. All participants completed all other cognitive and sensory tasks.

Prior to analyses, the data were checked to confirm when participants completed app-based tasks. All participants who completed the baseline survey did it on the same day as the onboarding session, as scheduled (n=42). Most participants completed the app-based task on the scheduled day however compliance fluctuated across later tasks. In total, 42 participants completed the Ishihara task (day 6), 36 and 37 completed TMT-A and B, respectively (day 31), 38 completed the spatial memory task (day 33), 37 completed Stroop, and 32 completed the audiometry task (day 45).

Data quality was also checked such as the number of errors and completion times of app-based tasks. Completion times included any time taken to read instructions and medians ranged from 36 to 111 seconds across all tasks except the audiometry task which had a median of 749 seconds (Supplementary Table 1). There were a few outliers in task time which may be due to technical errors or participants not closing the task immediately after finishing. However, responses and scores were all within the expected range except for one outlier in the app-based Stroop mismatch condition (Supplementary Table 2). Therefore, no cut-offs were placed on app-based task time. Due to the nature of the Corsi block test, all participants had a minimum of 2 errors. All participants successfully answered plate 1 (control plate) in the researcher-administered and self-administered versions of the Ishihara Color Deficiency Test. One participant’s responses were excluded from the Stroop correlations as they had 0 correct mismatch trials and had a mismatch time that was faster than their match trial time (Supplementary Table 2).

**Supplementary Table 2.**

*Task Completion Times in Seconds for All Self-administered Assessments, Including Time Taken to Read Instructions and Perform Pre-assessment Checks.*

| Task | Median (s) | Range (s) |
| --- | --- | --- |
| **Spatial Span** | **70** | **40 – 11306** |
| **TMT-A** | **38** | **20 – 50620** |
| **TMT-B** | **36** | **20 – 161** |
| **Stroop Test** | **111** | **81 – 879** |
| **Ishihara Color Deficiency** | **95** | **44 – 11735** |
| **dBHL Tone Audiometry** | **749** | **455 – 743262** |

**Supplementary Table 3.**

*Descriptive Statistics for Participant Errors Across Tasks from Pre-analysis Data Checks.*

| Task | Mean errors | Median | Range | Total possible trials |
| --- | --- | --- | --- | --- |
| TMT-A | 0.19 | 0 | 0-2 | 25 |
| TMT-B | 0.67 | 0 | 0-4 | 25 |
| Stroop dots | 0.02 | 0 | 0-1 | 24 |
| Stroop words | 0.05 | 0 | 0-1 | 24 |
| Stroop colored words | 0.55 | 0 | 0-5 | 24 |
| **Spatial Span** | **1.40** | **1** | **0-2** | **5** |
| **TMT-A** | **0.81** | **1** | **0-5** | **13** |
| **TMT-B** | **1.11** | **1** | **0-6** | **13** |
| **Stroop match** | **0.14** | **0** | **0-1** | **24** |
| **Stroop mismatch** | **2.62** | **1** | **0-24^** | **24** |

^ one participant had incorrect responses for all mismatch trials. Significant correlations were obtained including their data but also re-run excluding their data, which are presented in the results.

Note: self-administered, app-based tasks are highlighted in bold.

Bland-Altman Plots comparing each frequency and ear

**Supplementary Figure 1.**

*Bland-Altman Plots for Each Frequency of the Right Ear*
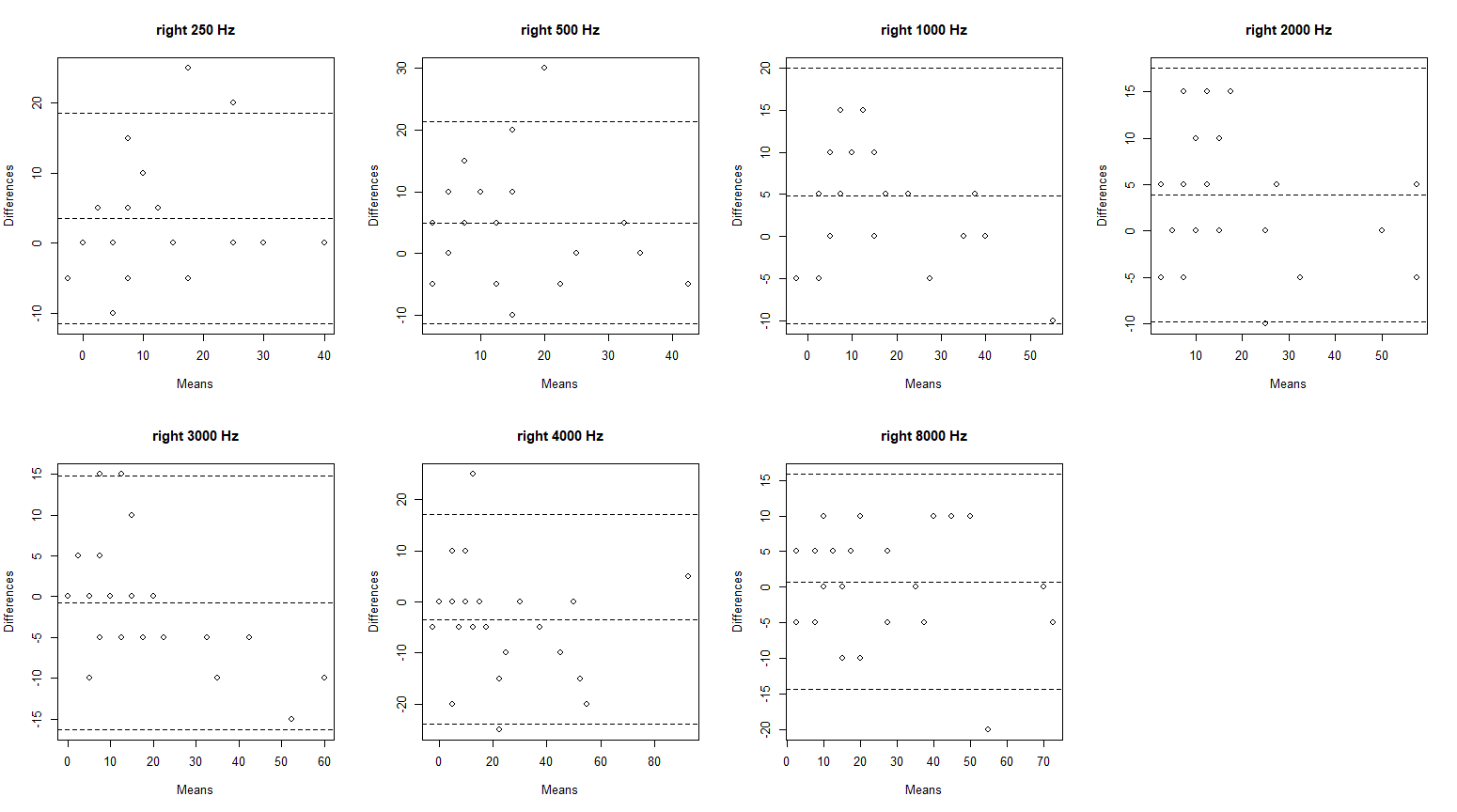


**Supplementary Figure 2.**

*Bland-Altman Plots for Each Frequency of the Left Ear
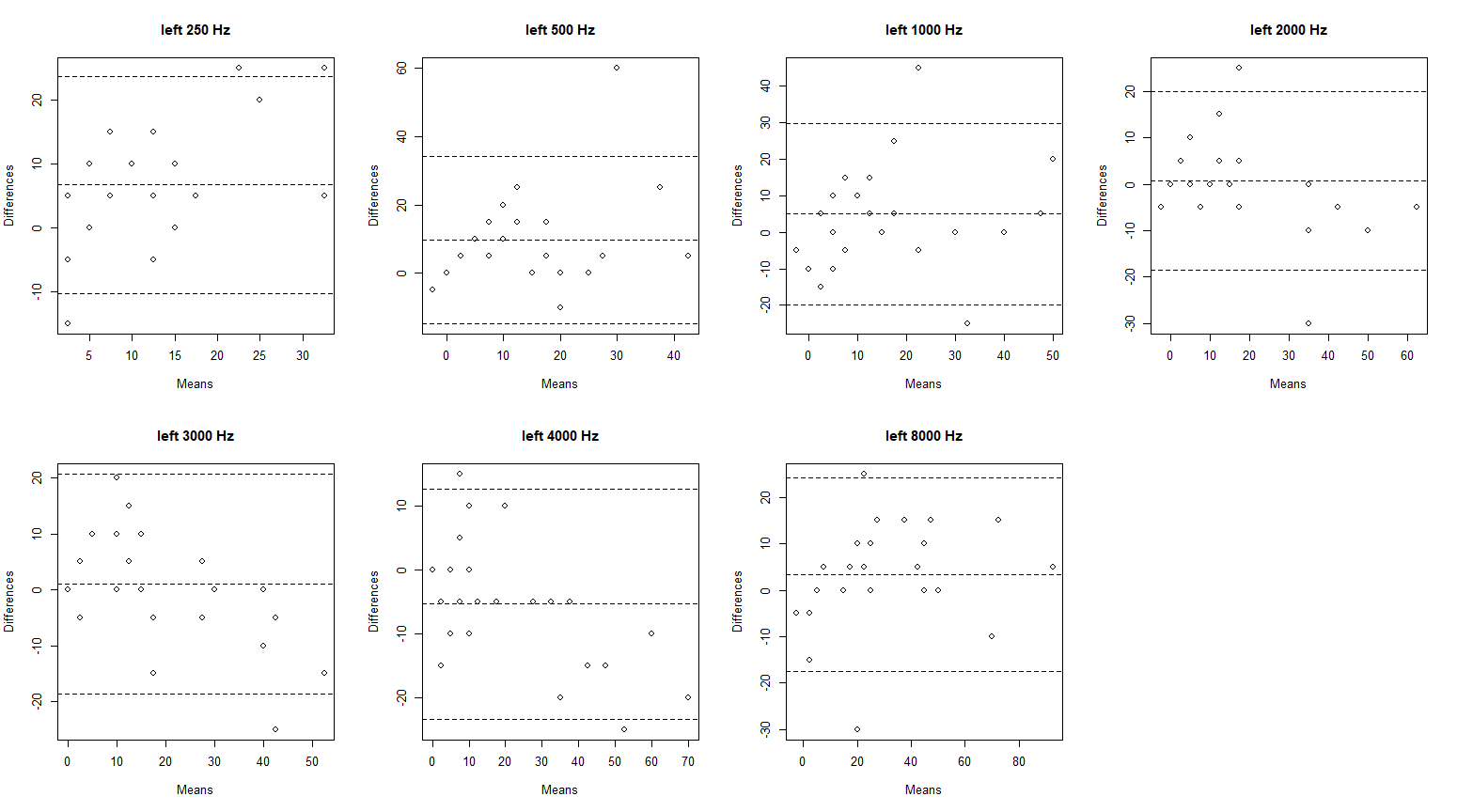
*
